# IBDDB: a manually curated and text-mining-enhanced database of genes involved in inflammatory bowel disease

**DOI:** 10.1093/database/baab022

**Published:** 2021-04-30

**Authors:** Farhat Khan, Aleksandar Radovanovic, Takashi Gojobori, Mandeep Kaur

**Affiliations:** School of Molecular and Cell Biology, University of the Witwatersrand, Private Bag 3, Johannesburg, Gauteng WITS-2050, South Africa; Computational Bioscience Research Center (CBRC), Biological and Environmental Science and Engineering (BESE), King Abdullah University of Science and Technology (KAUST), Thuwal, Jeddah 23955-6900, Kingdom of Saudi Arabia; Computational Bioscience Research Center (CBRC), Biological and Environmental Science and Engineering (BESE), King Abdullah University of Science and Technology (KAUST), Thuwal, Jeddah 23955-6900, Kingdom of Saudi Arabia; School of Molecular and Cell Biology, University of the Witwatersrand, Private Bag 3, Johannesburg, Gauteng WITS-2050, South Africa

## Abstract

To date, research on inflammatory bowel disease (IBD, encompassing Crohn’s disease and ulcerative colitis), a chronic complex disorder, has generated a large amount of data scattered across published literature (1 06 333) listed in PubMed on 14 October 2020, and no dedicated database currently exists that catalogues information on genes associated with IBD. We aimed to manually curate 289 genes that are experimentally validated to be linked with IBD and its known phenotypes. Furthermore, we have developed an integrated platform providing information about different aspects of these genes by incorporating several resources and an extensive text-mined knowledgebase. The curated IBD database (IBDDB) allows the selective display of collated 34 subject-specific concepts (listed as columns) exportable through a user-friendly IBDDB portal. The information embedded in concepts was acquired via text-mining of PubMed (manually cleaned and curated), accompanied by data-mining from varied resources. The user can also explore different biomedical entities and their co-occurrence with other entities (about one million) from 11 curated dictionaries in the indexed PubMed records. This functionality permits the user to generate and cross-examine a new hypothesis that is otherwise not easy to comprehend by just reading the published abstracts and papers. Users can download required information using various file formats and can display information in the form of networks. To our knowledge, no curated database of IBD-related genes is available so far. IBDDB is free for academic users and can be accessed at https://www.cbrc.kaust.edu.sa/ibd/.

## Introduction

Inflammatory bowel disease (IBD) is a complex intestinal disorder with two notable phenotypes: Crohn’s disease (CD) and ulcerative colitis (UC) (1). The general symptoms may include abdominal pain, severe cramping, weight loss, rectal bleeding and anaemia, growth retardation, etc. and is mainly diagnosed through colonoscopy. The location and nature of the inflammatory lesions inside the body differs, as CD affects the whole gastrointestinal tract, from the mouth to the anus, whereas UC primarily affects the colonic mucosa (2). Approximately 30% of patients with UC (3) and 70% of CD patients undergo surgical treatment (4). The incidence and prevalence of IBD are highest in westernized nations. Over one million people in the USA and 2.5 million in Europe are estimated to have IBD (5), and the incidence is believed to be increasing worldwide (6). Similarly, its prevalence is highest in Italy (322 per 100 000) followed by Canada (319 per 100 000) (7). Increased incidence of IBD, especially UC, has been found among sub-Saharan Africans (8). Population-specific differences have also been identified, e.g. in South Africa, the incidence of CD in 194 investigated patients was 18% (35) in whites, 78% (152) in mixed race and 4% (7) in blacks (9). IBD does not present itself as an isolated disease but exhibits several extraintestinal manifestations and complications. These manifestations include arthropathies, mucocutaneous, hepatobiliary, ophthalmological and peripheral arthritis (10, 11). There is also an increased risk associated with IBD patients for developing colorectal cancer (CRC) (12). Therefore, it is essential to investigate the role of different genes involved in IBD to decipher common pathways involved in the above-mentioned conditions and IBD.

Over the past few years, large-scale genome-wide association studies (GWASs) have made significant contributions in dissecting the genetic component involved in the development and progression of IBD (http://www.ibdgenetics.org/). Initial studies performed on thousands of patients have linked 99 IBD susceptibility loci to IBD and out of these, 71 related with CD, 47 with UC and 28 with both CD and UC (13). Another study identified >160 loci linked to IBD (14). Various cellular pathways including, but not limited to, apoptosis, autophagy, endoplasmic reticulum stress, oxidative stress and carbohydrate metabolism have been linked with IBD pathogenesis (15). Several signalling pathways have been linked with IBD [reviewed in (16)], along with metabolic alterations in serum (17), urine, faeces, etc. of IBD patients (18). A strong link is demonstrated between inflammatory pathways of IBD and the CRC as several molecular pathways contributing to the development of CRC have been identified (19). IBD patients have been reported to have 20 times increased chances of developing CRC than control population (20). Earlier studies have proposed an approximately 4-fold increased risk of progressing CRC in IBD patients (21–23), which has been confirmed using large-scale meta-analysis studies (24). The situation is worsened by poor prognosis of CRC in IBD patients (25). Henceforth, it is imperative to understand the molecular basis of IBD and to interrogate comprehensively the roles of genes implicated in IBD.

In the current era of big data, where a plethora of genetic, transcriptomic and genomic data are available, it has become crucial to analyse these data in connection with molecular and signalling pathways, protein–protein interactions, drug interactions, etc. to draw meaningful biological conclusions that can provide insights into the processes involved in the development and progression of a disease. These insights will also be useful for repurposing drugs for the treatment of related diseases. The above-mentioned insights can be effectively achieved if the required data and the related literature are available in a user-friendly format to the researchers. Since no such resource is currently available for investigating IBD, we have created a database of genes experimentally linked to IBD that will enable researchers to mine, combine and investigate IBD-related literature. We have integrated information from various publicly available resources along with manually curated list of IBD-related genes to create a fully searchable database with capabilities such as hypothesis generation and intelligent visualization of information in the form of networks. The database presented here will act as a one-stop shop for exploring the relevant IBD-related information from various perspectives. We hereby describe the construction and utility of the Inflammatory Bowel Disease Database (IBDDB).

## Methods

### Construction of the knowledgebase

To facilitate information exploration and association discovery, IBDDB contains combined data-mined information from several external data sources along with text-mined information using in-house Dragon Exploration System (DES) hosted by the King Abdullah University of Science and Technology (KAUST), which has been used to create several knowledgebases (KBs) referenced (references 141–159) in the latest publication (26) as well as for patent-pending discoveries based on text and data mining. The system has been shown to have high percentages of precision (81–100%), recall (96–100%) and F-measure (88–100%) for several dictionaries as explained in detail in our previous publications (27, 28).

The DES system functions based on two essential components: dictionaries, which are controlled vocabularies comprising concepts from specific fields, and the PubMed Records. A global index is created by matching the titles and the abstracts retrieved from PubMed against the dictionaries to create a KB index, which links each concept to its occurrences in the PubMed records. This allows highlighting of the concepts in the sentences along with allowing occurrence and co-occurrence counts. The KB index allows identification of enriched concepts and their association with each other, thus facilitating hypothesis generation. The enrichment refers to over-representation of the concepts or pairs of concepts in the KB as compared to the whole PubMed. This enrichment is characterized by the default false discovery rate of 0.05. KB generation is mostly an automated process, whereas the generation of the query for retrieving the relevant PubMed IDs, selection of relevant dictionaries and possible additional cleaning of dictionaries by elimination of promiscuous terms is performed manually (29).

### Concepts and dictionaries

The concepts contained in the KB are terms compiled in the dictionaries. In IBDDB, 11 dictionaries are used with the number of terms included in brackets [Biological processes (1112), Cellular component (276), Biological action (94), ChEBI (76 566), Dietary supplements (2707), Diseases (15 878), Drugs (13 582), Human Genes (42 061), Metabolites and enzymes (61 127), Pathways (3717), and Toxins (8407)].

Since one concept can be identified in various versions, to provide non-redundant information the concepts are normalized (i.e. only one index internally in DES would represent the concept that may appear in various versions of names, synonyms and symbols that would all describe the same entity). Therefore, concepts in all dictionaries are normalized. The sources used to compile dictionaries are listed (29). The dictionaries were cleaned manually to remove the ‘common’ English terms and by eliminating promiscuous terms based on the frequency of their appearance, so as to reduce the ‘noise’ in the DES reports.

DES was used to obtain a list of potential IBD-related genes by matching terms to 1 06 333 abstracts downloaded through PubMed search using query [(inflammatory bowel disease OR Crohns OR ulcerative colitis) AND (human OR mice OR mouse OR rat)], and this list was then manually curated. DES generated a list of potential 400 genes, out of which 208 were manually curated using strict criteria as explained below. To test if we identified a reasonable number of relevant genes through DES, we also tested a well-known text-mining tool PolySearch 2.0 (30). The keywords ‘Inflammatory Bowel Disease’, ‘Crohns Disease’ and ‘Ulcerative Colitis’ generated a potential list of 94, 18 and 0 genes/proteins, respectively, using PubMed and PubMed Central as resources. A total of 112 genes/proteins were found by PolySearch 2.0., and only 15 of these were verified via manual curation. DES generated a list of 400 genes, and out of these 208 were curated and verified and other 81 genes were curated while going through PubMed literature.

### IBD gene curation criteria and other unique attributes of IBDDB

All PubMed records found to be linked with initial set of 400 genes generated by DES were read manually (both abstract and full-text wherever available), and required information related to experimental validation was extracted. Several genes that were mentioned in the full-text and not covered in the abstract but were experimentally linked to the target diseases were also included in the list of the curated genes. We discarded genes that were just mentioned in the records and not investigated in the wet laboratory settings. Also, some genes mentioned in the PubMed literature were identified by using computational analysis but not verified in laboratory settings, so these were excluded as well.

Using manual curation, we found 289 genes experimentally linked with IBD. The inclusion of genes in IBDDB was based on the strict criterion that only genes proved to be experimentally linked with IBD in published literature in PubMed (PMID) (31) and PubMed Central (PMCID) (32) based on the experiments performed in human samples and animal models by one or more low-throughput experiments such as immunohistochemistry, RT-qPCR, western blot, ELISA, FISH, etc. be included. The genes based on tissue microarray and high-throughput RNA-Seq data were included if they were validated by using any of the above-mentioned low-throughput methods or genes that were found to be highly significantly differentially expressed as compared to normal/healthy samples were included. Genes reported to harbour IBD-linked single nucleotide polymorphism (SNPs) and their variants have been also included in the database. The selected and experimentally verified genes were further validated through GeneCards (https://www.genecards.org/) database by investigating various disease-related pathways, ontologies, etc.

We then supplemented the data by adding pre-compiled data-mined information on IBD (referred to as ‘concepts’, see Table 1), such as Transcription factor binding sites (TFBSs), Transcription factors (TFs), Transcription Start Site (TSS), drug interactions, disease interactions, pathways, fold change of expression in tissues, etc. Additionally, links to several external databases are provided, such as UniProtKB (33), OMIM ID (34), ENTREZ ID (35, 36), ENSEMBL ID (34, 37), HGNC GENE ID (https://www.genenames.org/) (38), Drug Gene Interaction database (DGIdb) (39), DrugBank (40), Drug Signature Database (DSigDB) (41), GeneCards (42), Refseq DNA sequence (34, 43), Chromosome location (44, 45), Kyoto Encyclopedia of Genes and Genomes (KEGG) pathways (46), Gene Ontology (GO) annotations (47) from Database for Annotation, Visualization and Integrated Discovery (DAVID) (48).

**Table 1.** Overview of the gene database columns

Column	Data
Id	Primary key
HGNC_gene_symbol	Gene symbols retrieved from a curated online repository of Human Gene Nomenclature Committee–approved gene nomenclature.
HGNC_gene_name	Human Gene Nomenclature Committee–approved gene names
chromosome_location	Chromosome segment location of human genes
snp_variants	SNP variants
IBD_phenotypes	Denotes the link of the genes to IBD and/or its two subtypes—CD and UC
previous_name	Provides the previous name and aliases used for IBD gene.
omim_id	The Online Mendelian Inheritance in Man Identifier
omim_diseases	The Online Mendelian Inheritance in Man diseases
uniprotkb	UniProtKB/Swiss-Prot KB, protein database identifiers
entrez_id	Entrez gene identifiers
HGNC_id	The HGNC identifier
ensembl_id	Ensembl identifier
refseq_DNA_sequence	RefSeq DNA sequence accession number
pmid	The PubMed reference number assigned by the NIH National Library of Medicine to abstracts indexed in PubMed database.
pmcid	The PubMed Central reference number assigned by the NIH National Library of Medicine to full-text papers.
experimental_evidences	The experimental information used as evidences for particular IBD-associated genes retrieved using PubMed publications
study_subject	The subject of the study where gene was verified: Human, mouse, rat and zebra fish
up_down_regulation	Expression associated with up or down regulation in the literature
inflamed_sites	The inflamed location of site in the gastrointestinal tract
tissues_samples	Tissue samples (colonic mucosa, saliva, etc.) collected from various inflamed sites
cell_lines	The cell lines used for experimental validation
literature_disease	Different diseases associated with this gene retrieved from literature during curation
biological_process	Biological process (GO) description retrieved from DAVID database
cellular_process	Cellular process (GO) description retrieved from DAVID database
molecular_function	Molecular function (GO) description retrieved from DAVID database
kegg_pathways	KEGG pathways
reactome_pathways	The reactome pathways downloaded from Enrichr database
DGIdb_interactiontypes	The DGIdb provides links between genes and their known or potential drug associations
dsigdb	The drug signatures database retrieved from Enrichr database
tfs_transfac	TRANSFAC database linked TFs downloaded from Enrichr database, which have binding sites in the promoter of the gene
tfs_chea	TFs downloaded from Enrichr database. The experiments such as ChIP-chip, ChIP-seq, ChIP-PET and DamID (the four methods referred to as ChIP-X) were used to profile the binding of TFs to DNA at genome-wide scale
tfs_encode	TFs collected by Encode Consortium. TFs were downloaded from the Enrichr database
tfs_opossum	TFs retrieved from Opossum 3.0., the web-based tool for the detection of over-represented conserved TFBSs in the sets of genes or sequences.

IBDDB provides information about the source and its associated reference for the data presented in each of the column.

IBDDB also provides information about the potential regulation of IBD genes by mapping the promoters of the curated genes with the TBSs. We have used different databases such as Enrichr (44), JASPAR (49) and TRANSFAC (50) and a web-accessible software oPOSSUM version 3.0 (51) to generate TFBSs. We extracted the conserved binding sites of TFs on the promoters covering regions (−1000 to +200) around TSS for 289 IBD genes. We used JASPAR mammalian matrix library to analyse the mapping of TFBSs to the promoter sequences using position frequency matrix model and scanned the position for each nucleotide base using weight matrix models.

### Text-mining module

**Figure 1.**
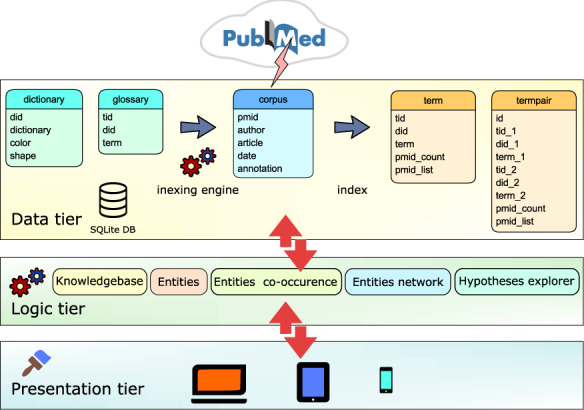
Text-mining module is based on 3-tier architecture. (i) Data tier database: dictionary table containing the list of dictionary names and the glossary table containing list of biomedical terms is applied by the indexing engine to the corpus table containing PubMed abstracts. Indexing module updates the corpus table and produces two new tables: term table, containing indices of terms and termpair table containing indices of co-occurring terms in the PubMed corpus. (ii) Logic tier is driven by ajax calls that access tables and combine data. (iii) Presentation layer is based on the jQuery/Bootstrap framework producing responsive web pages that can be viewed on a variety of devices.

Text-mining was performed on 106 333 IBD-relevant abstracts, which were downloaded from PubMed, and the parsed text was then loaded into SQLite database and indexed (Figure 1). The indexing engine used 11 dictionaries with 2 22 726 biomedical terms. The process generated an index of over 11 000 terms and close to 3 00 000 co-occurring terms. The terms were normalized by using dictionary of synonyms and homonyms. Use of synonyms is important for constructing correct term networks and hypotheses generation. For example, genes typically have several alternate names and symbols, which can cause unnecessary complex interconnections. Moreover, users exploring such networks can be misled into thinking that they are discovering new connections while viewing the same network but with different labels. Homonymous terms still represent challenge in text-mining, and they were not used extensively. When the offline processing is finished, the text-mining database is used as a data tier in the 3-tier architecture as illustrated in Figure 1. The logic tier consists of numerous modules serving ajax calls initiated from the user interface in the presentation tier. All data exchange is performed in JavaScript Object Notation (JSON) format, providing another layer of modularity. To support various devices and screen sizes, the presentation layer is built on jQuery/Bootstrap framework producing device responsive web pages.

## Results

### Capabilities of IBDDB

IBDDB combines the power of the manually curated information with text-mined data, which allows user to extract, visualize and dissect information (using explore and visualize tabs) with ease through an interactive user-friendly interface. The ‘Explore’ tab provides information listed in Table 2. The ‘Visualize’ tab allows the user to create networks of terms, providing advanced capabilities to merge and extract information provided in the PubMed abstracts. In the following sections, we will explain the key capabilities of network construction and hypothesis generation by creating examples.

**Table 2.** Different types of explorable modalities available in the ‘Explore’ tab of the IBDDB

Tab	Details
^a^Inflammatory Bowel Disease (IBD) Database	Curated database of 289 gene, contains 34 columns (concepts)
Knowledgebase	To explore the IBD-related titles and citations from PubMed literature. This includes sentences extracted from the literature, which show link to IBD
Biomedical entities	To explore biomedical entities linked to IBD by providing information about linked dictionary, frequency in the literature and the link to PubMed literature
Biomedical entities co-occurrence	Helps user to explore co-occurrence of biomedical entities linked to IBD. It allows to find co-mention of two entities in the published literature by selecting any two dictionaries (out of 11 listed). The relevant PubMed articles can also be retrieved for further exploration
Hypothesis explorer	Hypotheses explorer allows to identify new association among selected terms selected from different dictionaries (A–B and B–C are known and A–C are suggested links between biomedical entities)

### Network construction and visualization

**Figure 2.**
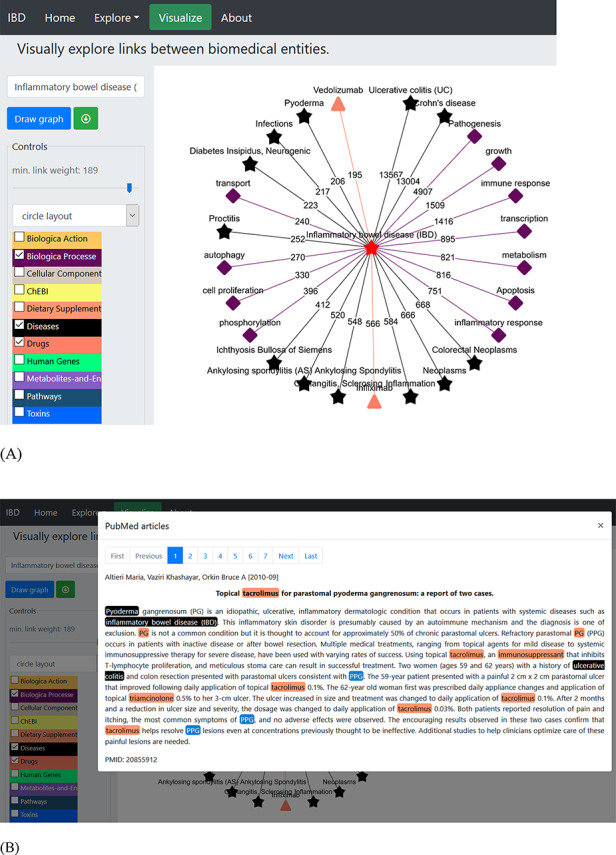
An example of a term-specific network. The network was generated using ‘inflammatory bowel disease’ as the search term, and the network represents the connections with terms from three different dictionaries (biological process, diseases and drugs). (A) The layout of the displayed network is circle with colour-coded nodes representing the colour of the dictionary they belong to. The digits on the edges represent the number of PubMed records, where co-occurrence of the two terms was found. (B) The PubMed literature appearing as the edge linking ‘pyoderma and inflammatory bowel disease’ was clicked. The terms are colour-coded based on the colour of the dictionary they belong to (shown in the left panel).

Working with a plethora of biomedical entities from various dictionaries can be a complex process. The ‘Visualization’ tab allows creation of networks among different entities based on their occurrence in the literature. This network uses Cytoscape (52) platform for visualization and has various built-in display layout (e.g. cola, circle, grid, concentric, breadthfirst and cose layout) options (as shown in Figure 2A). The user can build the network of choice by selecting one or more dictionaries at each step and trimming off the links not deemed useful. The nodes represent the entities from the selected dictionaries and are colour-coded, while numbering on the edges or links represents the number of PubMed records (Figure 2B) showing co-occurrence of the entities (shown by connected nodes). The user can download the constructed network in ‘.png’ file format along with PMID numbers of the abstracts.

### Example illustrating the capabilities of the IBDDB to generate novel hypothesis

Previously, altered lipid profiles have been linked to IBD, in particular low total plasma cholesterol levels and high triglyceride level (53). We were interested in further exploring the role of lipids (particularly cholesterol) in IBD and to find out hypothetically if these affect IBD-related conditions such as pyoderma gangrenosum (PG). PG is a skin ulcer disease found primarily in the immune system disorders and occurs at a higher frequency in IBD patients (54). For a better understanding of the hypothesis that we will generate in the following sections, it is important to comprehend that cholesterol is present intracellularly as well as extracellularly and is regulated by different cellular mechanisms at these locations (55). To prevent intracellular cholesterol accumulation, it is effluxed on to ApoA1 on high-density lipoprotein (HDL) for removal through reverse cholesterol transport to liver. When this process is halted or altered, low plasma cholesterol levels are found, which point to excessive intracellular cholesterol accumulation (56), leading to enhanced inflammation as cholesterol efflux pathways suppress the activation of inflammasome (57), resulting in chronic diseases such as atherosclerosis, obesity and IBD (53, 58, 59).

### Hypothesis generation

The hypothesis generator works on the basis of the principle proposed by Don R. Swanson in 1980s, stating that if ‘A’ is related to ‘B’, and ‘B’ is related to ‘C’, then ‘A’ may be related to ‘C’, and has been used to make several literature-based discoveries (60). DES reports indirect links between terms to generate potential hypothesis. For example, Disease A has highly expressed Protein B (one record) and second record documents association between Drug C showing inhibition of Protein B, then according to Swanson linking it may be hypothesized that Drug C may be tested to treat disease A.

**Figure 3.**
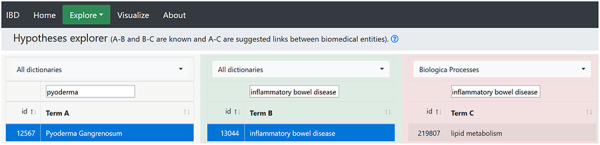
The hypothesis generated by the hypothesis explorer showing that PG may be linked to lipid metabolism.

By selecting ‘Pyoderma gangrenosum’ as ‘Term A’ and ‘Inflammatory bowel disease’ as ‘Term B’, we searched for ‘Term C’ by selecting biological processes dictionary, and 162 terms appeared, where lipid metabolism was a part of these terms (Figure 3). This may mean that lipid metabolism may play a part in PG. A PubMed search using keywords ‘Pyoderma gangrenosum AND lipid metabolism’ produced only one article (61). The article explained that in 94.8% of German PG patients, metabolic syndrome (hypertension, diabetes and lipid dysfunction) was recorded as a co-morbidity. Lipid metabolism dysfunction was recorded in 10.8% patients, CD in 4.5% and UC in 4.2% patients. This study provides support for our hypothesis and suggests further exploration of the hypothesis.

Since cholesterol levels were found to be reduced in IBD patients and cholesterol is a type of lipid, we decided to develop further connections between cholesterol and PG by creating a network of terms using the ‘Visualization’ tab.

**Figure 4.**
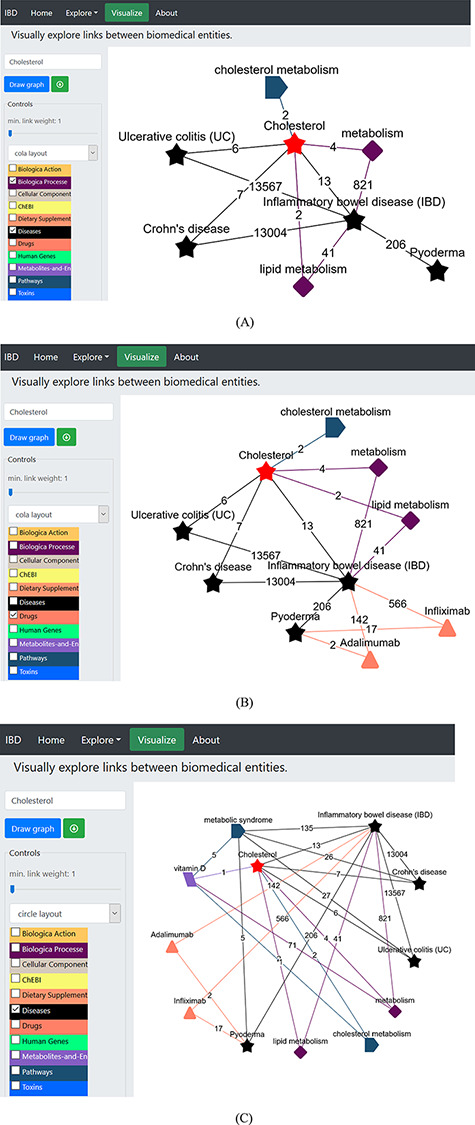
(A) Network construction to identify the direct relationship between pyoderma and IBD through PubMed abstracts. (B) Illustrates the construction of network to identify the link between pyoderma and IBD though common drugs used for treatment of both diseases. The orange triangles represent the ‘Drugs’ dictionary used to find drugs commonly used for treatment of both PG and IBD. (C) Illustrates the link between pyoderma and cholesterol through metabolic pathways. The red star represents the initial searched term ‘cholesterol’. The black stars represent the ‘Diseases’ dictionary. The purple-coloured diamond represents the ‘Biological Processes’ dictionary. The rectangular light purple colour represents the ‘Metabolites and Enzymes’ dictionary. The coloured edges represent the colours of their respective dictionaries. The number shown on each edge showcases the number of publications that link the associated nodes.

We began by selecting the ‘Visualize’ tab (Figure 4A) opening the network page, which is embedded with various dictionaries. Since we were interested in cholesterol and what role it plays in IBD and PG, we clicked on the ‘select term’ box on the left menu, typed the term ‘cholesterol’, deselected all other dictionaries except for the ‘biological process’, ‘diseases’ and ‘Pathways’ dictionaries listed in the left panel (Figure 4A), and pressed ‘Draw graph’ button (Figure 4A). This showed connections between cholesterol and lipid metabolism, cholesterol metabolism and IBD, among others. We then filtered the unwanted entities by selecting one or more nodes (shift + drag with left click) and right-click to delete, or we could limit the link by sliding the button placed the draw graph tab (Figure 4A). This can also be done by limiting the link (edge) weight to a number using the slider [e.g. ‘2’, which means only terms (nodes) that were found to co-occur in two or more records will be kept, while others below this number will be removed from the network]. Then, we unselected ‘Pathways’ dictionary and clicked the node ‘inflammatory bowel disease (IBD)’; this extended the network of terms linked with this node, and among these was ‘Pyoderma’ connected to the node via 206 records. We removed all other nodes from the network and got the network shown in Figure 4A. The relevant PubMed records demonstrating the association between entities can be accessed by clicking on the edge (link); this helps to validate the generated hypothesis using IBDDB. Figure 4A shows interconnections among different terms, and it is clear that cholesterol is linked to IBD, UC and CD, and is part of the lipid metabolism, which is linked with IBD. Therefore, there is a great possibility that cholesterol may be linked to ‘Pyoderma’ but no published record exists so far. Therefore, this is a hypothesis that will be explored further in the following sections.

In a UK-based study, the risk of death with PG was found to be three times more than that in general population, and 72% higher than IBD patients (62); we further expanded the network to find the connections among PG and IBD in terms of common drugs, genes and pathways involved along with identification of common therapeutic drugs. We selected the ‘drugs’ dictionary and clicked on ‘Pyoderma’ and then ‘inflammatory bowel disease’ terms on the network. After removing all other links except Adalimumab and Infliximab (Figure 4B), these drugs used in the treatment of IBD (Adalimumab and Infliximab) showed direct links with PG. These biologics are currently being prescribed as anti-TNF therapy as the first-line treatment to PG patients associated with or without IBD (63, 64). This demonstrates that the network generation in IBDDB is able to find known connections among different entities. The link between cholesterol and pyoderma is demonstrated through ‘pyoderma - metabolic syndrome - vitamin D – metabolism/cholesterol metabolism - cholesterol’. We uploaded ‘Pathways’ linked to pyoderma by selecting the relevant dictionary and deselecting all others and then further linking other dictionaries (diseases, metabolites and enzymes) to find an indirect connection between cholesterol and pyoderma (Figure 4C). This demonstrates that ‘cholesterol’ is linked to ‘pyoderma’ through metabolic pathways.

**Figure 5.**
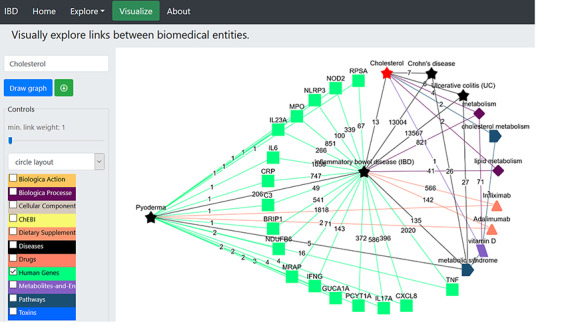
Illustration of the construction of network to identify the potential genes linked to pyoderma and IBD. The red star represents the searched term ‘cholesterol’. The green squares represent the ‘Human genes’ dictionary. The black stars represent the ‘Diseases’ dictionary, and the orange triangles represent ‘Drugs’ dictionary. The burgundy-coloured diamonds represent the ‘Biological Processes’ dictionary. The coral blue colour pentagon shape represents the ‘Pathways’ dictionary. The coloured edges represent the colours of their respective dictionaries. The number allocated on each edge showcase the number of publications that link to the associated nodes.

We further attempted to find common genes between PG and IBD and explored their potential link to cholesterol (Figure 5). We clicked the ‘Human Genes’ dictionary while deselecting all other dictionaries and then clicked on ‘pyoderma’ and ‘inflammatory bowel disease’ terms on the network. We found several genes to be common between PG and IBD (Figure 5). Sixteen genes were found to be common between PG and IBD. We explored the role of selected four genes IL23A, IL17A, TNF and Interferon gamma (IFNG) in IBD and PG using published literature. IL17A cytokines are linked to IBD, and their expression levels were found to be higher in early PG lesions along with Th 1-promoting transcription factors STAT1 and STAT4 (65). IL17A is an important factor that leads to the induction of autoimmunity-causing IBD (66). Furthermore, GWASs of IBD patients linked many SNPs in the IL23A receptor gene locus, asserting that IL23A plays an important role in IBD (67, 68) and PG (69) pathogenesis. Also, the abnormal T-cell responses and release of TNF-α, a potential pro-inflammatory cytokine, was found to cause PG pathogenesis (70).

The sharing of genes between PG and IBD is expected as these genes encode for either pro-inflammatory cytokines or regulate cytokines (71) and thus play a central role in inflammation-related processes, but we were interested in linking these genes with cholesterol, so that we can substantiate the hypothesis generated above. The IL23/IL17 axis plays a central role in psoriases (72), and TNF-α works synergistically with IL17 (73). The IL17-mediated inflammatory pathways have been found to be dependent on intracellular cholesterol accumulation in psoriasis, which is a chronic inflammatory skin disease (74). IFNG, a known suppressor of inflammation, has been found to mediate the downregulation of the sterol biosynthesis pathway (75). Intriguingly, it was demonstrated that the hydroxylated form of cholesterol mediates the negative-feedback pathway of IFN signalling, thus affecting IL1 family cytokine production and inflammasome activity (76). Additionally, growing experimental evidences have shown that hydroxycholesterols are vital as regulators of immune function; therefore, alteration of cholesterol content in plasma membrane can lead to antiviral, anti-inflammatory and pro-inflammatory effects (77). Since dysregulation of sterol metabolism contributes to inflammation and cholesterol accumulation in macrophage foam cells is known to induce inflammasome activation (78), it is critical to understand the role of cholesterol and lipid metabolism in PG.

The above-presented example demonstrates that the IBDDB has the capability to identify new potential interactions from the literature, which can provide understanding of the molecular pathways involved in inflammation-related diseases such as PG. Statins (cholesterol synthesis–blocking drugs), a class of cholesterol-lowering drugs, have been found to reduce risk of onset of IBD (79) and CRC (80), and prescription of statins for IBD prevention and treatment has been debated over the years (81, 82). The literature on the use of cholesterol-lowering drugs in PG does not exist in PubMed. This opens up possibilities to repurpose cholesterol-lowering drugs for treatment of PG, especially when cholesterol emboli have been identified as key reason for untreatable ulcers (83).

## Discussion

Despite the availability of thousands of records in PubMed, to the best of our knowledge not even a single database of curated IBD-associated genes exists so far. To fill this caveat, we have presented here the first IBD database (IBDDB) of experimentally verified IBD genes, which are sourced from PubMed records. Its user-friendly web interface allows the investigation of various features of IBD genes listed in the database and provides vital data necessary for in-depth evaluation of required genes at GO and pathway levels. IBDDB provides users with various tools to explore, investigate and visualize enriched and the most significant concepts (entities) from various dictionaries. It also allows to identify co-occurring terms to generate new hypotheses by providing connections between terms from published literature. The networks of the selected concepts can be gradually built and adjusted. We believe this database will save valuable time for researchers and clinicians and hope that it will facilitate the biological discovery process. IBDDB is a unique resource as it is a combination of curated and text-mined information, which is easily explorable and user-friendly. It is distinctive from other databases in several aspects: (i) This is the first manually curated database providing extended information about experimentally validated IBD-related genes; (ii) it provides pre-compiled biomedical text-mining information on IBD, which otherwise require complicated computational analyses; (iii) it integrates data on 34 concepts such as experimental techniques used to validate the role of a gene in IBD, sites inflamed in IBD, other diseases linked to the genes, tissue samples used for validation, molecular interactions, pathways, GO, gene expression level in mice or humans, TFs predicted to have binding sites on the promoters of IBD-implicated genes using four different tools, etc.; (iv) it contains data on drugs associated with IBD genes; (v) it has the hypothesis generation capability by creating networks of genes and other biomedical entities using 11 curated dictionaries; and (vi) it can present networks in six different layouts while data can be exported in various formats such as excel, csv and pdf, as well as printing. Despite these listed advantages, IBDDB has its shortcomings as far as the text-mined information is concerned. The text-mined information is restricted to electronic records only, whereas extracted concepts are limited by the completeness of the dictionaries, and the co-occurred terms may not entail meaningful association. Further advancement in the text-mining methodologies coupled with standardization of the information presentation in the publications would help to increase the accuracy of the text-mined data. Nonetheless, IBDDB can help create new potential interactions between terms and can help in the creation of new knowledge.

In summary, IBDDB allows information exploration through various searches where users can explore most significant entities or create links among different entities. It can also generate interesting hypotheses, create interactive networks and export results. We believe that IBDDB will be a very informative resource for researchers and clinicians.

## Future developments

IBDDB will be updated every 6 months as new literature is published. We will also continuously work to enhance the user interface as well as to implement improved text-mining capabilities. Other dictionaries will be compiled (e.g. nutrients and microbes) and integrated in order to extract extended information patterns.

## Data Availability

The data underlying this article are available at https://www.cbrc.kaust.edu.sa/ibd/. The data were derived from sources in the public domain and URLs can be accessed by clicking ‘?’ on top of each column in the database.
